# Supplementary material for: Causal Relationship between Aging and Anorexia Nervosa: A White-Matter-Microstructure-Mediated Mendelian Randomization Analysis
Source: Biomedicines. 2024 Aug 16;12(8):1874. doi: 10.3390/biomedicines12081874 (PMC11351342; doi:10.3390/biomedicines12081874)
Supplement: Supplementary file 1 [file biomedicines-12-01874-s001.zip › Supplementary Figure/Figure S7/Single SNP plot of MR analysis for ubm-b-1896 to AN.pdf]

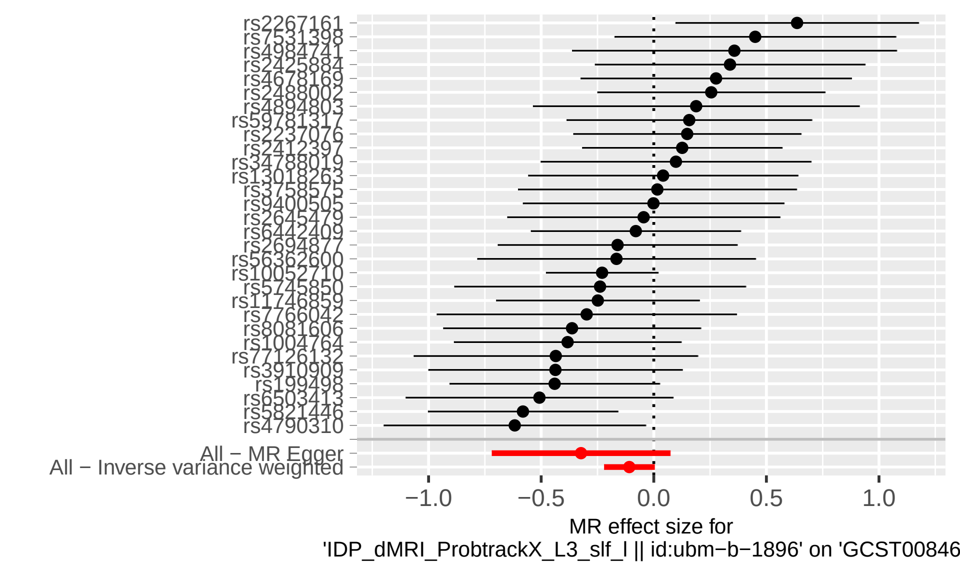
rs2267161
rs7531398
rs4984741
rs2425884
rs4678169
rs2488002
rs4894803
rs59781317
rs2237076
rs2412397
rs34788019
rs13018263
rs3758575
rs9400505
rs2645479
rs6442409
rs2694877
rs56362600
rs10052710
rs5745850
rs11746859
rs7766042
rs8081606
rs1004764
rs77126132
rs3910909
rs199498
rs6503413
rs5821446
rs4790310
All − MR Egger
All − Inverse variance weighted
−1.0
−0.5
0.0
0.5
1.0
MR effect size for
'IDP_dMRI_ProbtrackX_L3_slf_l || id:ubm−b−1896' on 'GCST00846
